# A simple cell-alignment protocol for sedimentation velocity analytical ultracentrifugation to complement mechanical and optical alignment procedures

**DOI:** 10.1007/s00249-018-1328-9

**Published:** 2018-08-29

**Authors:** Guy Channell, Vlad Dinu, Gary G. Adams, Stephen E. Harding

**Affiliations:** 1 National Centre for Macromolecular Hydrodynamics, The University of Nottingham, Sutton Bonington, LE12 5RD UK; 2 Queens Medical Centre, University of Nottingham, Nottingham, NG7 2HA UK; 3 Universitetet i Oslo, Postboks 6762, St. Olavs Plass, 0130 Oslo, Norway

**Keywords:** Protein aggregation, Dimerisation, Improving measurement precision

## Abstract

In establishing the sources of data variability within sedimentation velocity analysis in the analytical ultracentrifuge and their relative importance, recent studies have demonstrated that alignment of the sample cells to the centre of rotation is the most significant contributing factor to overall variability, particularly for the characterisation of low levels of protein aggregation. Accurate mechanical and optical alignment tools have been recently designed. In this study, we (1) confirm the effect of misalignment observed by others on the estimated amounts of bovine serum albumin (BSA) monomer and dimer, and the sedimentation coefficient value for the BSA dimer; and (2) demonstrate the high performance of a mechanical alignment tool and the usefulness of a simple and complementary enhanced manual alignment protocol which should be useful for situations where these tools are not available.

## Introduction

Sedimentation in the analytical ultracentrifuge has become a powerful matrix-free method for evaluating the molecular integrity and heterogeneity of macromolecules in the solution (see for example, Harding 2018 & references cited therein). A particularly important application is the use of sedimentation velocity in the ultracentrifuge for the establishment of the presence of aggregates or dissociation products in protein- and glycoprotein-based systems such as monoclonal antibodies (Lu et al. 2008), or the extent of dimerisation of proteins eliciting a T-cell response in glycoconjugate vaccines (Abdelhameed et al. 2012). A companion matrix-free technique for providing heterogeneity-related information is dynamic light scattering (Nobbmann et al. 2007). It has the advantage of being relatively fast, although not as resolving as analytical ultracentrifugation.

In establishing the sources of data variability from sedimentation velocity analysis in the analytical ultracentrifuge and their relative importance, Arthur et al. (2009) demonstrated that alignment of the sample cells to the centre of rotation is the most significant contributing factor to overall variability for the characterisation of low levels of protein aggregation. Working with a known irreversible monomer/dimer antibody mixture containing 3% dimer, they demonstrated the dimer peak is broadened and migrates with lower sedimentation coefficients with increased angle of misalignment when analysed by the *SEDFIT* algorithm (Schuck 2000; Dam and Schuck 2004). The monomer peak also showed broadening although the monomer sedimentation coefficient was unaffected. Arthur et al. (2009) considered in detail, ways of reducing the error caused by misalignment and also other factors such as numbers of measurements, temperature control, possible centrepiece variability, identifying the true meniscus precision and random and time-independent noise. Ways of improving precision were further considered in subsequent papers (see, e.g. Gabrielson et al. 2010, 2011), including focusing on a system of bovine serum albumin (BSA) monomers and dimers.

**Fig. 1:**
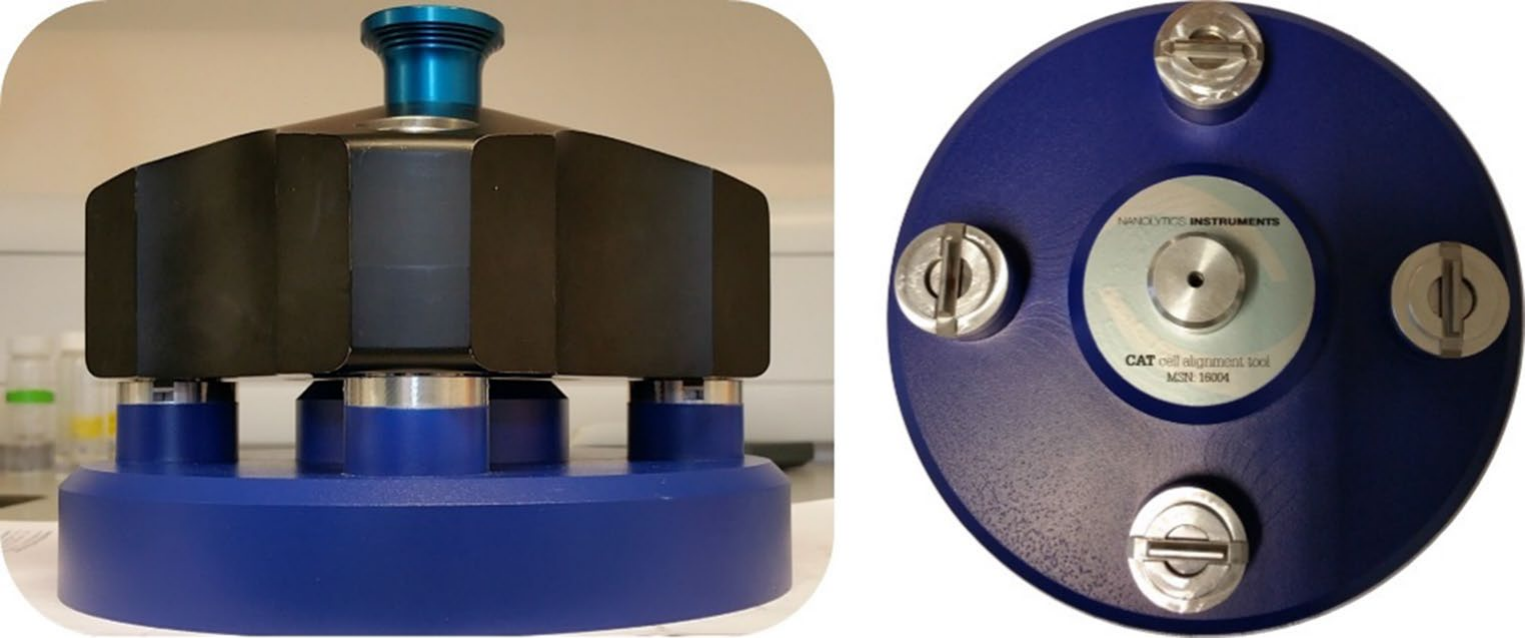
Nanolytics cell alignment tool. Courtesy of Dr. K. Schilling

For cell alignment, mechanical cell-alignment tools have been designed and are now commercially available from Nanolytics GmbH (Potsdam, Germany) (Fig. 1), and Spin Analytical (Berwick, ME USA). In addition, an optical alignment method of comparable precision—and the potential for even greater precision—has been developed by researchers at Eli-Lily (Doyle et al. 2017), but this is not yet commercially available. These researchers investigated the total amount of aggregate predicted from *SEDFIT* in a monoclonal antibody system, at misalignment angles ranging from − 2.5° to + 2.5° as a possible metric for alignment. Due to high uncertainty in quantification of aggregates, the minimum was hard to determine, and appeared not at 0° but still within ± 1°.

In this short study, we confirm the problems of misalignment observed previously, demonstrating the reproducibility of a mixing protocol which avoids cell assembly and re-assembly under potentially different conditions of window position and strain. We show not only the power of a mechanical alignment tool, but also the usefulness of a simple, inexpensive, enhanced manual alignment protocol with the aid of 10 × magnifying eye-piece and show it is possible to approach the performance of more sophisticated mechanical and optical alignment tools. This should provide a useful complementary approach for situations where these advanced alignment tools are not available.

## Materials and methods

### Alignment of cells

Cells were aligned with the Nanolytics mechanical cell-alignment tool or manually with the aid of a simple 10× magnifying glass.

### Misalignment tool

**Fig. 2:**
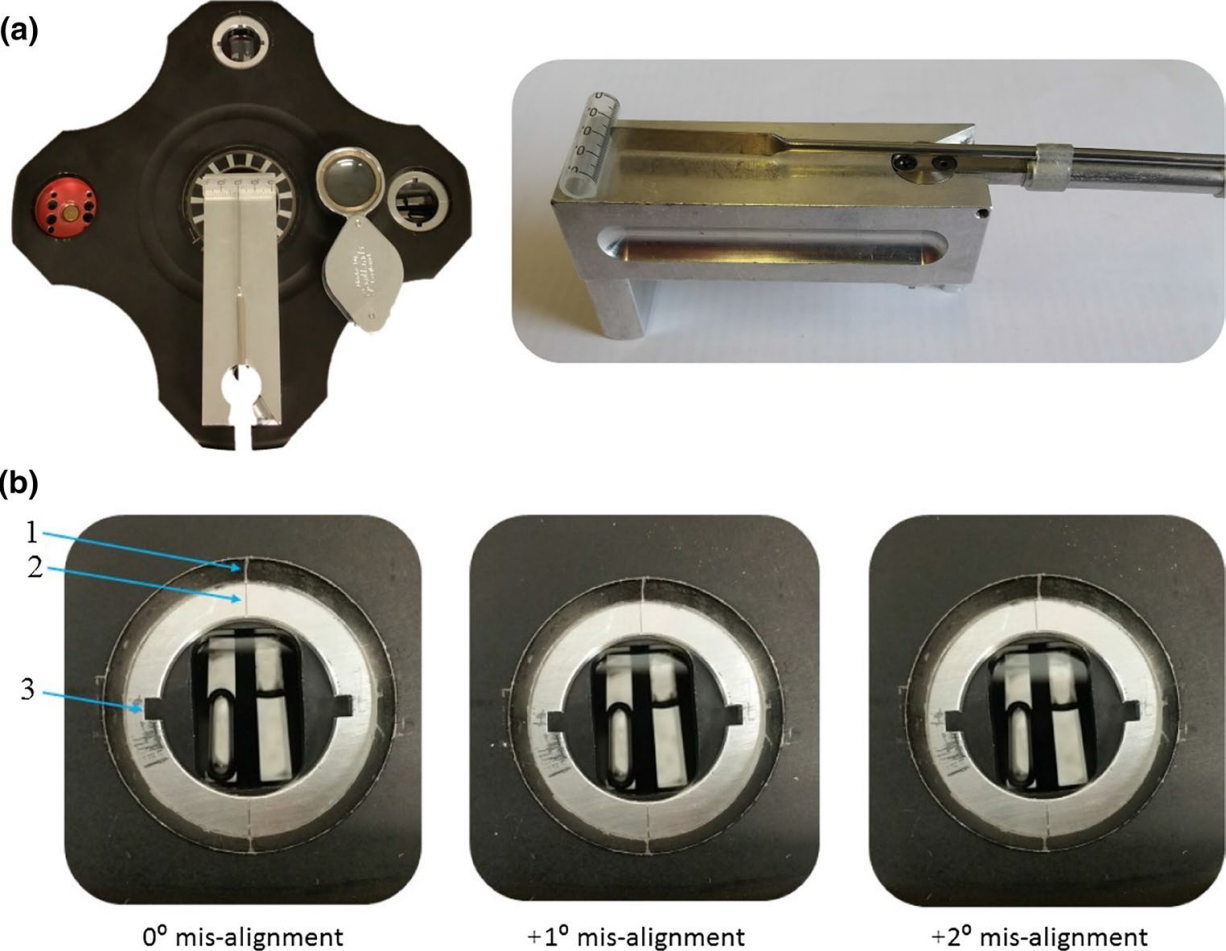
Cell misalignment tool. **a** In position at the base of the rotor, **b** cells after misalignment. 1,2 alignment lines, 3 locating groove

An in-house constructed misalignment tool, Fig. 2, was used to set a range of off-set angles (up to 4°), shown in Fig. 2, after initial alignment by the Nanolytics instrument.

### Solutions

A solution of bovine serum albumin at a concentration of 0.7 mg/mL in phosphate–chloride buffer (pH 6.8, *I* = 0.10) was used. Samples were run in a four-hole rotor using unused Beckman carbon-filled Epon 12 mm double sector centrepiece cells at a rotor speed of 45,000 rpm for 5 h at 20.0 °C under a sedimentation velocity protocol using absorbance and interference optics. To ensure a consistent comparison with regards to cell tightening/stress on the cell components, after sedimentation the cells were removed from the rotor and re-dispersed using a roller mixer protocol for 20 min, ready for the next alignment angle run. Data were analysed using a c(s) model within the *SEDFIT* program (Schuck 2000; Dam and Schuck 2004) employing a translational frictional ratio *f/f*_0_ = 1.25 (where *f* is the frictional coefficient of the protein and *f*_0_ is the corresponding value for an anhydrous spherical particle of the same mass and volume) in the c(s) model.

### Re-dispersion protocol

To validate the re-dispersion protocol and confirm the absence of effects due to possible pelleting, the 0° off-set sample was run in triplicate, two consecutive runs and a third at the end of the misalignment series. A fourth run using a 0° manually aligned cell, with only the cell and rotor indexing marks for alignment, was also conducted as a direct comparison against the mechanical (Nanolytics) tool. In addition, the following misalignment angles were investigated after initial alignment with the Nanolytics tool: + 1°, + 2°, + 4°, − 1°, − 2°, − 4°.

## Results and discussion

### 0° work: effect of re-dispersion

**Fig. 3:**
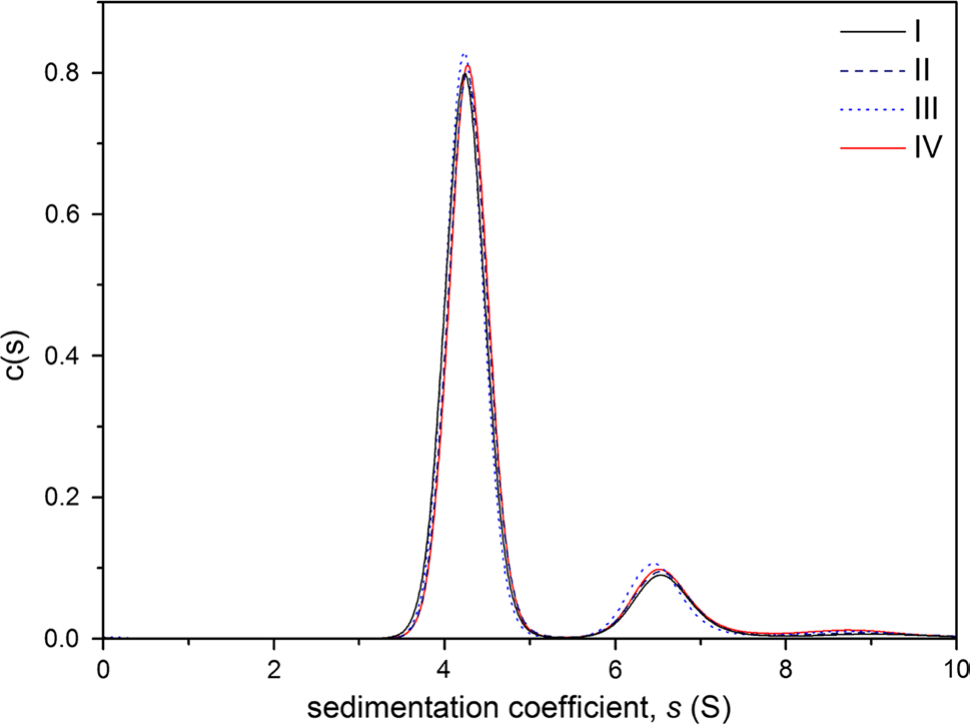
Mechanical tool aligned 0° data. Sedimentation coefficient distribution plot for 0.7 mg/mL bovine serum albumin in phosphate–chloride buffer (pH 6.8, *I* = 0.1). Alignment with Nanolytics mechanical tool set for 0°. I: 1st run. II: 2nd run after roller re-dispersal. III. 3rd run at 0° after completion of all the misalignment and re-dispersal experiments. IV: Enhanced manual alignment, 0°

SEDFIT c(s) vs s analysis showed complete consistency between the first 0° off-set experiment, the second experiment after re-dispersal and the third run at the end of the misalignment series (Fig. 3). Encouragingly, this confirmed complete re-dispersal. The two primary peaks are in good agreement with the expected molecular weight for the monomer and dimer of BSA (Fig. 3). From the areas under the peaks it is possible to estimate the proportions of monomer and dimer relative to each other, and again there is a close agreement between the runs.

### Effect of misalignment

**Fig. 4:**
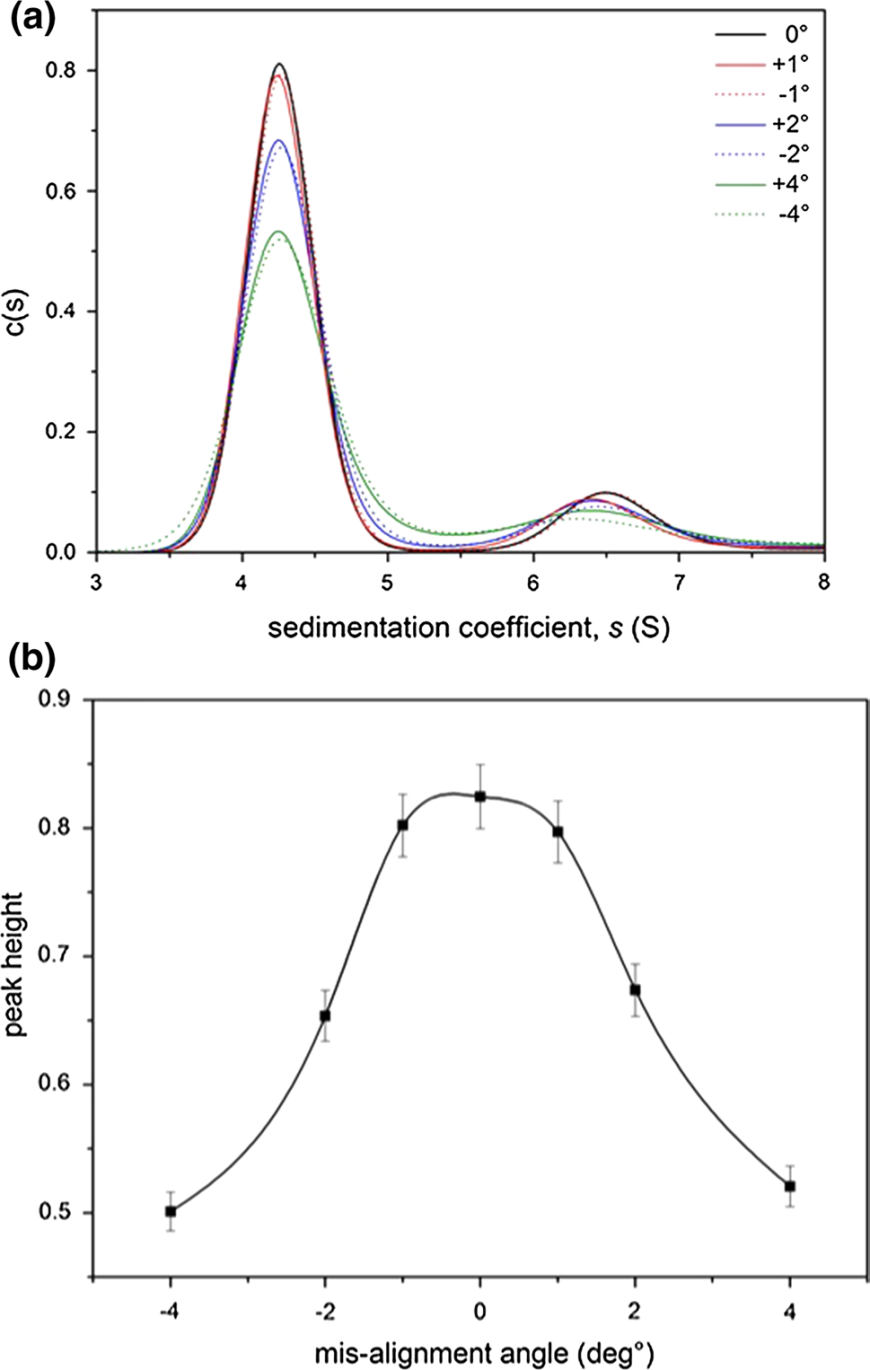
Effect of misalignment. **a** Sedimentation coefficient distribution plot for 0.7 mg/mL bovine serum albumin in phosphate–chloride buffer (pH 6.8, *I* = 0.1) for a range of misalignment angles. **b** Monomer peak height as a function of misalignment angle. The average value and standard error for 0° is shown and is assumed by % the same for the misaligned angles

**Fig. 5:**
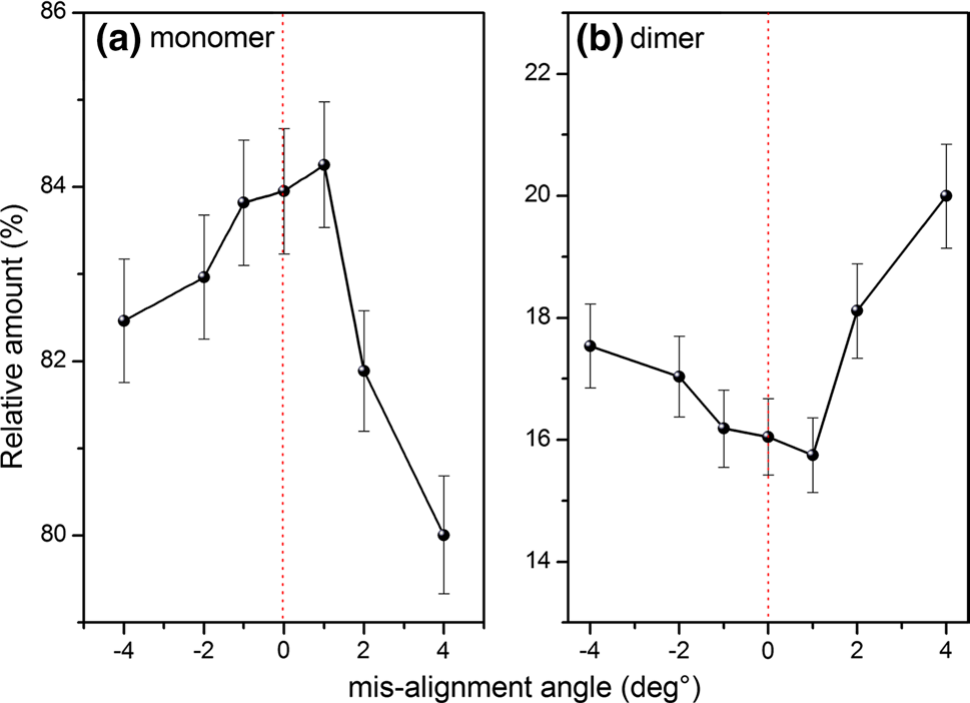
Relative percentages (%) of estimated BSA monomer and dimer as a function of misalignment angle. The average value and standard error for 0° is shown and is assumed by % the same for the misaligned angles

The effect of increased misalignment angle is clearly shown to cause an increased peak broadening for both the monomer and dimer whereas only the dimer shifts to lower sedimentation coefficients (Fig. 4a), which is consistent with the studies of Arthur et al. (2009) and Gabrielson et al. (2011). Plotting peak height against misalignment angle shows a consistent height reduction through increments in both +ve and −ve values of off-set angle in a symmetrical form (Fig. 4b). Using again the relative amounts of monomer and dimer, it can be seen that the minimum is not at zero (a similar observation was made by Doyle et al. (2017), where they were using total aggregate amount as the metric) (Fig. 5).

### Comparison of Nanolytics instrument aligned with enhanced manual alignment

Finally, on comparing the 0° samples aligned using the Nanolytics (Potsdam, Germany) mechanical alignment tool with our very best 0° “enhanced” manual alignment using only the cell and rotor indexing marks, with the aid of an enhanced manual procedure (with the 10 × magnifier), we observed the amounts of monomer and dimer to be very close to those found by the mechanical tool (Table 1 and line IV of Fig. 3). With the tool, a mean value of 82.0% was found for the relative amount of monomer and 18.0% of dimer. With the enhanced manual method, values of 84.2% for monomer and 15.8% for dimer were obtained. These data would suggest that it is possible to approach the performance of the mechanical alignment tool with the enhanced manual method.

**Table 1:** Relative percentages of monomer and dimer after three roller re-dispersions (I–III) with the mechanical alignment tool, and (IV) with the enhanced manual method

Sample	Monomer %	Dimer %
I BSA 0° off-set run 1	81.62	18.38
II BSA 0° off-set run 2	81.79	18.21
III BSA 0° off-set run 3	82.67	17.33
IV BSA 0° off-set, enhanced manual method	84.17	15.83

## Concluding remarks

From this simple study we can confirm earlier observations that cell alignment has a significant effect on the broadening of peaks when processed using a c(s) model in *SEDFIT.* For multicomponent systems this can lead to peak-overlap which can compromise the analysis. The data in this study would suggest that if we align the AUC cells using manual alignment with the aid of 10 × magnifying eye-piece, it is possible to approach the performance of the mechanical alignment tool. Beyond this level of alignment precision, other limitations in the instrumentation such as temperature control and accuracy of data capture can become more significant limitations. As the accuracy in these areas also improves (Zhao et al. 2014), optical alignment procedures and the great precision they offer (Doyle et al. 2017) will then become increasingly significant.

## References

[CR1] Abdelhameed AS, Morris GA, Adams GG, Rowe AJ, Laloux O, Cerny L, Bonnier B, Duvivier P, Conrath K, Lenfant C, Harding SE (2012). An asymmetric and slightly dimerized structure for the tetanus toxoid protein used in glycoconjugate vaccines. Carbohyd Polym.

[CR2] Arthur KK, Gabrielson JP, Kendrick BS, Stoner MR (2009). Detection of protein aggregates by sedimentation velocity analytical ultracentrifugation (SV-AUC): sources of variability and their relative importance. J Pharm Sci.

[CR3] Dam J, Schuck P (2004). Calculating sedimentation coefficient distributions by direct modeling of sedimentation velocity concentration profiles. Methods Enzymol.

[CR4] Doyle BK, Budyak IL, Rauk AP, Weiss WF (2017). An optical alignment system improves precision of soluble aggregate quantitation by sedimentation velocity analytical ultracentrifugation. Anal Biochem.

[CR5] Gabrielson JP, Arthur KK, Stoner MR, Winn BC, Kendrick BS, Razinov V, Svitel J, Jianmg Y, Voelker PJ, Fernandes CA, Ridgeway R (2010). Precision of protein aggregation measurements by sedimentation velocity analytical ultracentrifugation in biopharmaceutical applications. Anal Biochem.

[CR6] Gabrielson JP, Arthur KK, Stoner MR, Winn BC, Kendrick BS, Razinov V, Svitel J, Jiang Y, Voelker PJ, Fernandes CA, Ridgeway R (2011). Measuring low levels of protein aggregation by sedimentation velocity. Methods.

[CR7] Harding SE (2018). The Svedberg Lecture. 2017. From nano to micro: the huge dynamic range of the analytical ultracentrifuge for characterising the sizes, shapes and interactions of molecules and assemblies in Biochemistry and Polymer Science. Eur Biophys J.

[CR9] Lu Y, Harding SE, Rowe AJ, Davis KG, Fish B, Varley P, Gee C, Mulot S (2008). The effect of a point mutation on the stability of IgG4 as monitored by analytical ultracentrifugation. J Pharm Sci.

[CR10] Nobbmann U, Connah M, Fish B, Varley P, Gee C, Mulot S, Chen J, Zhou L, Lu Y, Shen F, Yi J, Harding SE (2007). Dynamic light scattering as a relative tool for assessing the molecular integrity and stability of monoclonal antibodies. Biotech Gen Eng Rev.

[CR11] Schuck P (2000). Size distribution analysis of macromolecules by sedimentation velocity ultracentrifugation and Lamm equation modeling. Biophys J.

[CR12] Zhao H, Balbo A, Metger H, Clary R, Ghirlando R, Schuck P (2014). Improved measurement of the rotor temperature in analytical ultracentrifugation. Anal Biochem.

